# Age-Related Differences in Dietary Intake and Nutritional Status Among Older Adults in Croatia: Results from a National Food Consumption Survey

**DOI:** 10.3390/epidemiologia7030071

**Published:** 2026-05-21

**Authors:** Lidija Šoher, Daniela Čačić Kenjerić, Martina Pavlić, Dunja Ćosić, Ana Ilić, Ivana Rumbak, Jasna Pucarin-Cvetković, Darja Sokolić

**Affiliations:** 1 Department of Food and Nutrition Research, Faculty of Food Technology Osijek, Josip Juraj Strossmayer University of Osijek, Franje Kuhača 18, 31000 Osijek, Croatia; lidija.soher@ptfos.hr; 2 Centre for Food Safety, Croatian Agency for Agriculture and Food, Ivana Gundulića 36b, 31000 Osijek, Croatia; martina.pavlic@hapih.hr (M.P.); darja.sokolic@hapih.hr (D.S.); 3 Independent Subdepartments, Faculty of Food Technology Osijek, Josip Juraj Strossmayer University of Osijek, Franje Kuhača 18, 31000 Osijek, Croatia; dunja.cosic@ptfos.hr; 4 Department of Nutrition, Food Quality and Safety, Faculty of Food Technology and Biotechnology, University of Zagreb, Pierottijeva Ulica 6, 10000 Zagreb, Croatia; ana.ilic@pbf.unizg.hr (A.I.); ivana.rumbak@pbf.unizg.hr (I.R.); 5 Department of Environmental and Occupational Health and Sports Medicine, Andrija Štampar School of Public Health, School of Medicine, University of Zagreb, Rockefeller St. 4, 10000 Zagreb, Croatia; jasna.pucarin@snz.hr; 6 Division for Environmental Health, Croatian Institute of Public Health, Rockefeller St. 7, 10000 Zagreb, Croatia

**Keywords:** national survey, nutritional status, dietary intake, older adults, food categories

## Abstract

Background/Objectives: Understanding nutrient intake and diet quality in older adults is essential for promoting healthy ageing and quality of life. The aim of the study was to assess dietary intake and nutritional status in two age groups of older adults in Croatia (65–74 years and ≥75 years). Methods: A total of 786 participants aged 65 and older were included in this cross-sectional study. Data from the National food consumption survey (OC/EFSA/DATA/2017/01), based on the EU Menu methodology, were used. Data collection included a general questionnaire, the International Physical Activity Questionnaire, two 24-h recalls or food diaries, and anthropometric measurements. The effects of body mass index and physical activity level on dietary intake were analysed using a general linear model. Results: 21.5% of older adults in Croatia had a normal weight, while 78.5% of were classified as overweight or obese. Significant differences were recorded in energy and macronutrient intake between the two age groups. Body mass index was significantly associated with energy (kcal/day), fat intake (g/day), and intake of the meat, poultry, fish and eggs food group in the 65–74 year age group. In the ≥75 year age group, physical activity level showed an effect on energy, carbohydrates, and milk and dairy product intake. Intake of nutrient-dense foods and fluids was below recommendations in both observed groups. Conclusions: The study results, based on a representative sample, provide the first overview of the nutritional status of older adults in Croatia. These findings offer a foundation for public health initiatives and further research on the nutritional status of the older population in Croatia.

## 1. Introduction

Life expectancy in developed countries exceeds 70 years, and between 2015 and 2050, the proportion of individuals aged over 60 years is projected to increase from 12% to 22% globally [1,2,3]. According to the final results of the 2021 Population Census, Croatia is among the countries with an evident trend in population ageing. Individuals aged 65 years and older accounted for 22.5% of the total population, representing a significant increase compared with 16% in 2011 [4,5]. Such a demographic shift may represent one of the most significant demographic and public health challenges of the 21st century, particularly in the context of chronic disease prevention and nutritional care [6]. Increased longevity is often accompanied by higher morbidity and health deterioration, accounting for approximately 35% of the disease burden in high-income countries, primarily due to non-communicable and other degenerative diseases [7].

Ageing, as a natural biological process, is accompanied by a decline in the function of multiple physiological systems. Reduced taste and smell perception, impaired vision, decreased mobility, loss of muscle mass, and frailty can impair everyday functioning and lead to decreased appetite, altered food choices, and reduced ability to prepare and consume meals [1,4,8]. Malnutrition and age-related anorexia were among the primary nutritional concerns in this population. However, increased life expectancy, high obesity prevalence among individuals aged ≥65 years, and the cumulative health impact of long-term obesity have shifted this perspective [8]. Obesity, as a chronic and complex disease, has become one of the most significant global health challenges. In older adults, overweight and obesity are associated with reduced physical activity, inadequate dietary intake, previously mentioned age-related declines, and reduced quality of life [9]. A particular challenge in this population is sarcopenic obesity, a condition in which muscle mass and strength loss coexist with an increased proportion of adipose tissue, creating a higher risk for adverse health outcomes [10]. Alongside population ageing, chronic non-communicable diseases remain the leading causes of mortality. Monitoring obesity trends is therefore essential, particularly among older adults [11].

Ageing is economically more sustainable when older adults maintain good health and remain socially active. This largely depends on their health status, making healthy ageing a key public health priority. Although life expectancy is increasing, a longer lifespan does not necessarily translate into better health outcomes or healthier dietary habits [2]. Research indicates that older adults in European countries often have inadequate protein and dietary fibre intake, while intakes of sodium, saturated fat, and sugar exceed recommendations. In addition, research by Azzolina et al. (2020) shows that less than half of older adults in European countries achieve the recommended daily intake of fruit, vegetables, and legumes [12]. As energy requirements decline with age while nutrient needs remain stable or increase, older adults require diets with a higher nutrient density [7,13]. Dietary risk factors and physical inactivity are among the main contributors to the global disease burden [14]. Monitoring dietary habits in vulnerable populations is of particular importance. Understanding nutrient intake and diet quality in older adults is therefore essential for promoting healthy ageing and quality of life [13]. However, data on dietary intake and its association with body mass index (BMI) and physical activity (PA) across different age groups of older adults are very limited, especially for those over 75 years of age. Based on a nationally representative sample, the aim of this study was to assess dietary intake and nutritional status in two age groups of older adults (65–74 years and ≥75 years) in Croatia, with the hypothesis that BMI and PA significantly influence dietary intake and nutritional status in older adults.

## 2. Materials and Methods

The National Food Consumption Survey on adolescents and adults from 10 to 99 years of age (NIPNOD; OC/EFSA/DATA/2017/01), conducted from 2018 to 2023, provided harmonised data on dietary habits. The survey was conducted in accordance with the EU Menu methodology [15] and was approved by the National Ethical Board of Institute for Medical Research and Occupational Health (Class: 01-18/20-02-2/1; RegNo: 100-21/20-18; approval date: 23 September 2020). A detailed description of the study design, sampling procedure, data collection, and dietary assessment methods has been published previously [16].

### 2.1. Study Population and Data Collection

The survey encompassed 1913 adolescents, adults, and older adults across six Croatian regions. Based on the database of the Ministry of Internal Affairs, the study population was stratified by region, sex, and age, and evenly distributed across all four seasons. Potential participants were randomly selected according to predefined criteria to obtain a representative sample. Hospitalised individuals and those living in institutionalised settings were excluded from the study, except for older adults residing in nursing homes. All participants were informed about the aim and methods used in the study and provided written informed consent. To assess the nutritional status and dietary intake of older adults (65–74 years; ≥75 years), data collected on 786 participants were analysed.

Data collection was conducted through face-to-face, video, or telephone interviews, depending on the participants’ abilities. For data collection, NutriCro^®^ 3.0 software was used. The software included a general questionnaire on sociodemographic information and lifestyle factors, the International Physical Activity Questionnaire (IPAQ), and two 24-h recalls or food diaries per participant. In the case of elderly participants, a food diary was used. In addition, anthropometric data were obtained through direct measurement when possible or were self-reported. All data collection was carried out by trained interviewers with backgrounds in nutrition and food science.

### 2.2. Dietary Intake Assessment

Dietary intake was assessed using the average of two non-consecutive 24-h dietary recalls or food diaries, distributed across all weekdays and all four seasons. For older participants who were able to recall their dietary intake, multi-pass 24-h recalls were administered. The interviewers were trained to review the list of food and beverages with the participants al least three times. For participants with limited recall ability, food diaries were used. Trained interviewers provided detailed instructions to record all foods, beverages, and food supplements consumed, without modifying their usual dietary habits. Consumed amounts and detailed information were collected, including preparation methods and producer information. Dietary supplement intake was recorded during dietary assessment, but it was not included in this analysis. When a food diary was used, participants were provided with a printed food diary form and a picture book. After the food diary was completed, the recorded information was collected and entered into the dietary assessment software. The NutriCro^®^ 3.0 software incorporates a food consumption database that includes a national food composition database [17], individual food items, composite dishes, and commercial products. For portion-size determination, a validated picture book [18], several picture sets from the PANCAKE study [19], standard portion sizes, and household measures were incorporated into the software. When these options did not allow accurate quantification, a free-text option was used. All data were entered and reviewed by trained interviewers.

Collected dietary intake data were classified into 13 main food groups, with additional sub-groups (Table S1). The main food groups included: grains, grain products and potato; fruit; vegetables; legumes, nuts and seeds; meat, poultry, fish and eggs; milk and dairy products; fats and oils; salty snacks; sweets; water and beverages; alcoholic beverages; dietetic products and miscellaneous foods.

### 2.3. Anthropometric Measurement

Participants’ body weight (kg) and height (cm) were measured during the initial face-to-face interview using a digital scale (Seca 877^®^, Hamburg, Germany) and a portable stadiometer (Seca 217^®^, Hamburg, Germany), respectively. When direct measurements were not feasible, anthropometric data were self-reported by participants. BMI was calculated as body weight in kilograms divided by height in square metres. Nutritional status was assessed according to World Health Organization [20] BMI cutoff values for adults and classified into the following categories: underweight (<18.5 kg/m^2^), normal weight (18.5–24.9 kg/m^2^), overweight (25.0–29.9 kg/m^2^), and obese (≥30.0 kg/m^2^).

### 2.4. PA Assessment

PA was assessed using a validated short form of the IPAQ [21]. The questionnaire assessed participants’ physical activity over the previous seven days. Through interviews, participants reported frequency and duration of walking, moderate-intensity, vigorous activities, and time spent in sedentary activities. Overall activity levels were estimated according to IPAQ scoring protocols and categorised into three levels: low, medium, and high [22].

### 2.5. Data Analysis

The data were organised as categorical and continuous (interval) variables. Standard descriptive statistics were used to describe the results. Categorical variables were presented as frequency and percentage, and median and interquartile ranges (IQRs) were used to describe continuous variables. Misreporting of energy intake, including both underreporting and overreporting, was assessed using the Goldberg cutoff approach, as described in EU Menu methodology [15], based on the ratio between reported energy intake and estimated basal metabolic rate. The normality of the distribution was tested using the Shapiro–Wilk test. As most variables were not normally distributed, non-parametric tests were applied. To test differences between two groups, the Mann–Whitney test was applied. Chi-squared analyses were used to compare dichotomous variables such as sex, place of residence and supplement use. The general linear model (GLM) included PA level and BMI, as well as their interaction (ACTIVITY and BMI), in order to assess their effect on dietary intake. Homogeneity of variances was tested using Levene’s test, and Bonferroni correction was applied for multiple comparisons between PA levels. For all tests, the level of significance was set at *p* < 0.05. Data analysis was conducted using the statistical software package Statistica (version 14.0.1.25, 1984–2020 TIBCO Software Inc., Hamburg, Germany), Microsoft Excel 2016 (version 16.0.5413.1000, 2016 Microsoft Corporation, Redmond, WA, USA), and IBM SPSS Statistics (version 26.0, IBM Corp., Armonk, NY, USA).

## 3. Results

Sociodemographic, lifestyle, and anthropometric characteristics according to age groups (65–74 years; ≥75 years) are presented in Table 1. The study included 786 participants with a similar sex and region distribution. Participants in the older age group (≥75 years) more frequently lived in urban settings (77.2%; *p* = 0.013), had a significantly higher proportion of widowed participants (50.9%; *p* ≤ 0.001), and a significantly lower proportion of smokers (5.8%; *p* = 0.001). No significant differences were observed in household income, education levels, or dietary supplement use between the two groups.

**Figure 1:**
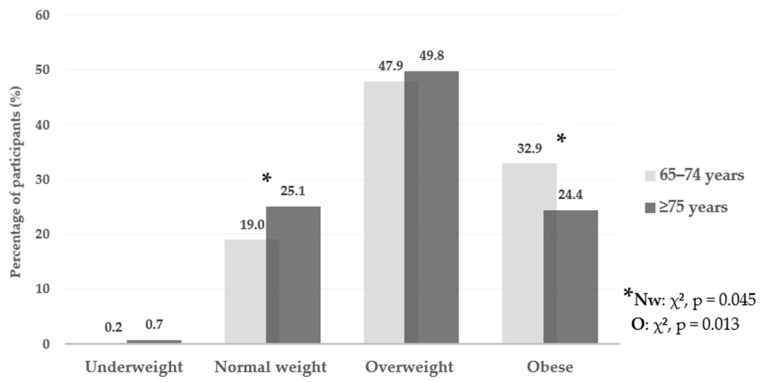
BMI categories distribution by age group in older adults (* significant differences between age groups were observed for the normal weight (χ^2^, *p* = 0.045) and obese category (χ^2^, *p* = 0.013)).

PA level was lower in the older group, with a higher percentage of participants with low PA levels (71.3% vs. 63.0%; *p* = 0.003). Anthropometric parameters differed significantly across all three parameters. All parameters were higher in the 65–74-year age group, including body weight (*p* < 0.001), height (*p* = 0.003), and BMI (*p* = 0.003). Overall, 78.5% of older adults in Croatia were classified as overweight or obese. Nutritional status, based on BMI in both groups of older adults, is presented in Figure 1.

The distribution of BMI categories according to the age groups revealed differences in the percentage of participants with normal body weight and obesity. In the ≥75 years age group, a higher percentage of participants had normal body weight (25.1%; *p* = 0.045), whereas in the 65–74 year age group, a higher percentage were classified as obese (32.9%; *p* = 0.013).

Significant differences in energy and macronutrient intake between the two age groups are summarised in Table 2. Participants aged 65 to 74 years had a significantly higher daily intake of energy (1628.9 kcal/day vs. 1442.9 kcal/day; *p* < 0.001), carbohydrates (170.2 g/day vs. 157.2 g/day; *p* = 0.001), protein (60.0 g/day vs. 53.4 g/day; *p* < 0.001), and fat (64.7 g/day vs. 55.3 g/day; *p* < 0.001). Regarding the contribution to daily energy intake, participants in the older group (≥75 years) had a higher energy contribution from carbohydrates (45.6% vs. 42.1%; *p* < 0.001), whereas participants aged 65–74 years had a higher contribution from fat. Protein contribution to daily energy intake did not differ between the two groups.

GLM analysis (Table 3) indicated that BMI may significantly predict energy and macronutrient intake in the 65–74 years age group. Energy (kcal/day) and fat intake (g/day) were significantly related to BMI, where higher BMI is related with higher energy (*p* = 0.048) and fat (*p* = 0.001) intake, while PA level and their interaction did not show a significant main effect. Regardless, post-hoc analysis showed higher energy (*p* = 0.010) and fat intake (*p* = 0.001) in participants with a low PA level in comparison with medium and high PA levels. As the assumption of homogeneity of variances was not met, these findings should be considered exploratory and interpreted with caution, as the reliability of the estimated effects and *p*-values may be reduced. In addition, BMI showed a significant effect on macronutrient energy contribution, where higher BMI was related to a lower energy contribution from carbohydrates (*p* = 0.014) and a higher contribution from fat (*p* = 0.003). Neither the PA level nor its interaction with BMI showed a significant effect on macronutrient contribution to energy intake in this group. In the older group (≥ 75 years), GLM analysis showed significant results in energy (*p* = 0.011) and carbohydrate intake (*p* = 0.009). PA level showed an effect on energy intake, where participants with a low activity level had a significantly higher energy intake compared to those with a medium or high activity level. For carbohydrate intake, BMI, PA level, and their interaction showed a significant effect. Estimated daily carbohydrate intake across PA levels, adjusted for BMI, indicated higher carbohydrate intake among low-active participants (203.9 g/day) compared with moderately and highly active participants (159.9 g/day; 159.6 g/day).

In the total study sample, the highest median daily intakes were observed for water and beverages (1028.0 g/day); grains, grain products and potato (209.9 g/day); fruits (160.0 g/day), milk and dairy products (153.0 g/day); vegetables (133.9 g/day) and meat, poultry, fish and eggs (129.5 g/day) (Table 4). A comparison between two age groups showed that participants aged 65–74 years had significantly higher intakes of meat, poultry, fish and eggs (*p* < 0.001), fats and oils (*p* = 0.003), sweets (*p* = 0.020), water and beverages (*p* < 0.001) and alcoholic beverages (*p* < 0.001). In contrast, participants aged ≥75 years had a significantly higher intake of milk and dairy products (*p* = 0.003). Although consumption of legumes, nuts, and seeds was low in both groups, participants aged 65–74 years had a significantly higher intake (*p* = 0.033). No statistical difference was observed in intake of grains, fruits, or vegetables between the age groups.

GLM analysis (Table 5) showed a significant effect of PA level on alcohol intake (*p* = 0.041) in the 65–74 age group. While post-hoc analysis did not show a significant effect, it revealed a trend of higher alcohol consumption in highly active participants. For meat, poultry, fish, and egg intake, BMI was a significant predictor (*p* = 0.005), where higher BMI correlated with higher intake. The main effect of PA level, as well as interaction of BMI and activity, did not show a significant effect. Similarly, BMI was significantly associated with consumption of processed meats (*p* = 0.007). Despite the post=hoc results showing a higher intake of processed meats in low-activity participants, the results should be interpreted with caution. In the ≥75-year age group, GLM analysis showed significant effects of BMI (*p* = 0.006), PA level (*p* = 0.018), and their interaction (*p* = 0.029) on milk and dairy product intake. Milk and dairy product intake varies according to PA level, with participants with a low level of activity consuming more than those who were highly active. Moreover, in participants with low PA levels, higher BMI is associated with lower dairy consumption. Fruit intake was also significantly influenced by BMI (*p* = 0.001), PA level (*p* = 0.006), and their interaction (*p* = 0.009). The interaction indicates that among low-active participants, those with a higher BMI consume less fruit.

## 4. Discussion

Based on the national food consumption survey on adolescents and adults from 10 to 99 years of age [16] and conducted on a representative sample in accordance with the EU Menu methodology [15], this study provides a first comprehensive overview of the nutritional status and dietary intake among older adults (>65 years) in Croatia, with a focus on age-related differences.

As expected, with an increase in age, there is a significant increase in the percentage of widows and widowers (24.5% in the 65–74 year age group vs. 50.9% in the ≥75 year age group). According to a meta-analysis by Besora-Moreno et al. (2020) [23], being single, widowed, or divorced is shown to be one of the most significant risk factors for developing malnutrition in the observed population. Losing a significant other and living alone may lead to unhealthier dietary choices and less enjoyment in preparing and consuming meals [23]. Results indicate that 67.4% of older adults in Croatia live in urban areas. Living in urban areas may be connected with healthier dietary choices, such as more integral bread consumption or adding less salt. Meanwhile, older adults living in rural areas had a higher risk of being malnourished [23,24]. Low income and low level of education may be other predictors of less healthy nutritional status according to the literature [23,25]. Although sociodemographic factors were not the main focus of this study, no significant differences were observed in education level or income between age groups. Regarding dietary supplements, no statistically significant difference was found between age groups, with supplements being used by around 37.8% of participants. This prevelence is comparable to the data from Begium, where 38.3% of the population reported supplement use, but higher than percentages reported for Portugal (26.6%) and Hungary (around 28%). Overall, supplement use appears common across Europe and tends to increase with age [25,26]. In addition to PA and poor dietary habits, smoking is one of the significant risk factors in developing chronic diseases. Our results showed a positive and significant decrease in the percentage of smokers according to age groups, from 13.1% in the 65–74 years group to 5.8% in the ≥75 year age group. This trend may be explained through several different aspects: older adults already have medical diagnoses and have changed their habits, or it could be related to less favourable socioeconomic status [24,25,27].

In the presented population, a low level of PA increased with age, from 63.0% participants with a low PA in the 65–74 age group to 71.3% in the older group. Previously presented results in the Croatian population indicated a higher prevalence, with 83.3% of participants over 65 years being physically inactive [28]. Retirement provides an opportunity to adopt healthier physical activity habits, although it is often associated with a decline in PA. Level of PA, along with other factors, may increase the risk of sarcopenic obesity. A decrease in highly active participants, from 15.7% to 5.1% in our sample, additionally confirms less PA with age and increased risk of losing physical functionality [29].

Overall, 78.5% of older adults in Croatia were classified as overweight or obese (BMI ≥ 25.0 kg/m^2^). In 2013, the general prevalence of overweight and obesity in Europe was 79.5%, with obesity increasing in most European countries, while overweight prevalence remained stable from 2005 to 2013 [11]. Similar results have been reported for neighbouring country Slovenia, where 74.0% of older adults (65–74 years of age) were overweight or obese (BMI > 25.0 kg/m^2^) [27]. A global meta-analysis by Khaleghi et al. (2025) found an obesity prevalence of 33.6% among older adults in Europe, which is consistent with the 32.9% observed in the 65–74 age group [9]. A significant decrease in average BMI was recorded with age. Although this trend may be viewed as positive in the general population, in older adults, it is generally viewed as an indicator of sarcopenia and muscle mass loss [25,29,30]. Therefore, the fact that results show an increase in participants with normal weight with age (from 19.0% in the 65–74 age group to 25.1% in the ≥75 age group) does not necessarily indicate better health, but may reflect an increased risk of nutritional inadequacy. In the observed population, body composition analysis and muscle mass assessment may be more accurate indicators of nutritional status [25,31].

Energy requirement decreases by 10% between the ages of 51 and 75, with an additional 10% reduction for each subsequent decade [28]. Consequently, there is no surprise that participants in the younger group (65–74 years) consume significantly higher amounts of energy in comparison to participants in the older group. There is a trend toward reporting a lower energy intake in the older population [13,25,27,32,33,34], which is consistent with our findings, as underreporters accounted for 24.1% of the total study sample. Even though energy requirements are lowering with age, the question remains whether low energy intake in older adults can cover all micronutrient needs that remain the same or become higher with age [4]. In addition to factors influencing lower food consumption, there is always a possibility of underreporting, which can be increased in this population due to age or, as some studies show, BMI. Australian research in older women showed that people with a higher BMI were more likely to under-report their energy intake [35]. A lower energy intake is accompanied by a lower macronutrient intake. Interestingly, a higher contribution to energy intake from carbohydrates was recorded in the ≥75 year age group, whereas in younger group (65–74 years) there was a higher contribution from fat. Compared to Croatian guidelines for nutrition in the older adults [4,28], results showed a higher than recommended contribution of fat in both age groups (35.2–37.7%), whereas carbohydrates were below recommendations (42.1–45.6%). Similar results for the contribution of fat to energy intake were reported in other countries [27,33,36]. A continuously higher proportion of fat contribution in older adults, in the context of reduced energy requirements and lower PA, may lead to increased energy intake and contribute to the development of obesity [32,36]. Protein intake in older age presents an important factor for lean (muscle) mass preservation and prevention of functional decline [36]. Recommendations on protein intake may differ, from 0.8–1.0 g/kg to newer ones from 1.0 g/kg to up to 1.5 g/kg. For contribution to energy intake on average, the recommendations are 10–20%, depending on the source [4,13,27,29,36]. The obeserved decrease in protein intake between age groups may lead to the aforementioned loss of muscle mass and functional decline. Despite the observed decrease in protein intake in grams, the contribution of protein to total energy intake remained around 15% and within the recommendations. In comparison, in Slovenia, most older adults meet the minimum protein recommendations but not higher recommendations (1.0 g/kg), while in Spain, physically active older adults had a higher protein intake [27,37]. The presented results did not show the effect of BMI and PA on protein intake, but they did show that participants aged 65–74 years with a higher BMI had higher energy and fat intake, as well as a higher contribution of fat to total energy intake. A positive correlation between fat contribution and risk of obesity, in particular sarcopenic obesity, was previously reported by Lee et al. (2021) [38]. A chronically high fat intake contributes to a chronic inflammatory state and further decrease in lean mass. Coupled with excessive energy intake, this creates a vicious circle of lower basal metabolic rate and adipose tissue accumulation in the older population [29,36,38]. In addition, research by Hansen et al. (2013) confirmed that people with obesity spend a larger amount of time in sedentary activity compared to persons with normal weight [39]. Significant nutritional imbalances, such as caloric intake exceeding requirements and macronutrient imbalances combined with low PA, contribute to a positive energy balance and increase the risk of obesity in this population [29,38].

With increasing age, there is a smaller amount of food consumed, as reflected in the presented results and differences between the two age gropus. The reasons for lower food intake can be numerous in older adults, from biological and physiological changes, reduced appetite due to disease or pain, impaired dental health, gastrointestinal conditions affecting chewing, swallowing or digestion, decreased sense of smell, taste and vision, to social and socioeconomic factors [12,28,40]. Some foods, such as legumes, nuts, and seeds, may require longer preparation times, greater chewing ability, or be harder to digest, which can further limit their consumption at this age. Economic constraints may also contribute since nuts and seeds are often relatively expensive. Altogether, this may promote a consumption of softer foods, often with a lower nutrient density [25,28,40]. This may explain the changes in the intake of the legumes, nuts, and seeds food group in the ≥75 group. Some of the challenges when it comes to dietary habits of older adults in Europe stem from an inadequate intake (<400 g/day) of fruits, vegetables, and legumes [12,25,27,41], a lower intake of key micronutrients and fibres [7], higher intake of energy-dense and nutrient-poor food [25,27,41], and inadequate fluid intake [25,42]. Similarly, in Croatia, the median intake of fruits and vegetables was 133.9 g/day and 160.0 g/day, respectively, which is lower than the recommended amount (<400 g/day). These results are in accordance with Belgian results, where 95% of older adults do not consume enough fruits and vegetables [41]. Increasing adherence to fruit and vegetable recommendations may be crucial in this population, as its protective role has been shown in preserving cognitive functions, as well as reducing the risk of chronic diseases and all-cause mortality [43,44]. Older adults with obesity are less likely to consume fruit and vegetables regularly, while physically active individuals are more motivated to follow a healthy diet [45]. Our results showed a similar trend, where in the ≥75 age group, low-activity participants with a high BMI consumed significantly less fruit. This suggests that physical inactivity may strengthen the negative association between higher BMI and diet quality in the oldest adults.

A higher intake of meat has been shown in previous studies in Central Europe, where intake of meat is often over the recommended amount [24,25,27]. Further analysis showed that a higher BMI is directly linked with a higher consumption of meat, poultry, fish, and egg group, as well as cured meat products in the 65–74 age group. High meat and cured meat consumption in Croatia is closely linked to the tradition of producing products in households and on family farms. Products such as kulen, sausages, prosciutto, and bacon are a part of the everyday diet, especially in older, more traditional individuals [46]. This tradition was also evident in younger population, where processed meats contributed the most to protein and fat intake in school-aged children with obesity [47]. Grains, grain products, and potatoes formed the dietary base, with a median intake of 209.9 g/day [25]. There needs to be a more detailed analysis to determine which grain products are most often consumed, with recommendations clearly suggesting restriction in refined grain intake [28]. Intake of sweet food may decrease with age, as recorded in our study, but sweets and refined grains often serve as an affordable and easily accessible source of energy in the older population. However, increased consumption may contribute to the development of obesity, diabetes, and cognitive decline [28,42]. Higher consumption of milk and dairy was recorded in the older group (≥75 years; 175.0 g/day), which may be the result of the aforementioned factors that emerge with older age. An increase in milk and dairy product consumption is positive, as it contributes to protein intake and represents an important source of calcium, despite intake remaining below recommendations (two to three servings) [28]. Additional factors influencing consumption of milk and dairy in this group are BMI and PA, where those with low activity and a higher BMI had a lower intake of milk and dairy. Dairy products are often avoided due to the misconception that they cause weight gain, but studies suggest they have a protective effect against weight gain [48].

Adequate fluid intake is an important component of the nutritional status of older adults. Insufficient intake and dehydration are related to cognitive and physical impairments, reduced appetite and thirst sensation, and constipation at this age. Even though fluid intake is commonly low within this population [25,27,49], a concerning decline was recorded in beverage intake between two age groups, which may further impact the aforementioned impairments [42]. Water represents around 66% of beverage intake in both observed groups, with the median intake being 717.5 g/day in the 65–74 age group and 592.9 g/day in the ≥75 age group. The recommended daily intake is set at 2 L of unsweetened beverages [28], including water from food, which neither group met. Intake of alcoholic beverages also declines between age groups. Similar to Slovenian adults [27], in Croatia, participants in the 65–74 age group consume more alcoholic beverages than those in the ≥75 age group. Also, participants with higher activity levels had a higher consumption of alcohol in this age group. Even though higher physical activity may suggest a healthier lifestyle in this age group, it may be related to more frequent social interactions. Moderate alcohol consumption, physical activity, and social interactions may improve quality of life in older adults [50].

In addition to providing valuable results in the older population in Croatia, the main strength of the study is the use of a national representative, stratified sample, and year-round data collection. The standardised methodology [15] ensures consistency and comparability of the results. This is also the first overview of nutritional status and dietary intake in older adults in Croatia. It has been underlined that there is a lack of research on nutrition-related factors in older adults [2], and the study therefore provides baseline data for future research and more regular studies. On the other hand, several key limitations need to be considered. The cross-sectional design does not allow conclusions about causal relationships between variables. The use of self-reported data can lead to reporting errors, which are common in nutrition studies. A major limitation is the underreporting of dietary intake, identified in 24.1% of participants, which may have led to lower estimates of energy intake. BMI was used as the sole anthropometric indicator; however, it may not adequately reflect body composition or nutritional status in older adults, particularly in cases such as sarcopenic obesity. Therefore, future studies should include more comprehensive assessments of body composition to improve the evaluation of nutritional status in this population [31]. Physical activity was assessed using the IPAQ short form, which, although validated, may overestimate physical activity and underestimate sedentary time in older adults at the individual level; however, it remains appropriate for use in large population-based studies such as this one [51]. In addition, although information on dietary supplement use was recorded, it was not included in this study, which may have influenced the estimated overall nutrient intake. A more detailed analysis of supplement use and its contribution to nutrient intake is planned for future research. Overall, the analysis serves as a foundation for understanding the challenges and further research of healthy ageing in Croatian older adults. The observed differences between the two age groups highlight the importance of tailored nutritional approaches in older populations.

## 5. Conclusions

This study demonstrated a high prevalence of overweight and obesity (78.5%) among older adults in Croatia, accompanied by decreased physical activity. Although energy and macronutrient intake declined with age, fat contribution to total energy intake exceeded recommendations, while intake of nutrient-dense food and fluids remained below recommendations in both observed groups. The presented results emphasise the importance of targeted public health initiatives focused on improving access to nutrient-dense foods, promoting adequate fluid intake, and regular physical activity, particularly resistance exercise, to preserve functional capacity and promote healthy ageing in this vulnerable population.

## Figures and Tables

**Table 1:** Participants’ sociodemographic and anthropometric characteristics according to age.

	Total (N = 786)	65–74 Years (n = 511)	≥75 Years (n = 275)	*p* ***
**Sex, n (%)**				0.899
Male	394 (50.1)	257 (50.3)	137 (49.8)	
Female	392(49.9)	254 (49.7)	138 (50.2)	
**Age, mean (sd)**	73.5 (6.1)	69.9 (2.8)	80.3 (4.4)	**<0.001**
**Region, n (%)**				0.093
Dalmatia region	147 (18.7)	99 (19.4)	48 (17.5)	
Istria, Primorje, and Gorski kotar region	107 (13.6)	69 (13.5)	38 (17.5)	
Lika and Banovina region	63 (8.0)	38 (7.4)	25 (9.1)	
Northern Croatia region	126 (16.0)	93 (18.2)	33 (12.0)	
Slavonia region	138 (17.6)	87 (17.0)	51 (18.5)	
Zagreb region	205 (26.1)	125 (24.5)	80 (29.1)	
**Place of residence, n (%)**				**0.013**
Urban	530 (67.4)	330 (64.6)	200 (72.7)	
Rural	251 (31.9)	179 (35.0)	72 (26.2)	
Unanswered	5 (0.7)	2 (0.4)	3 (1.1)	
**Employment status, n (%)**				**0.007**
Retirement	761 (96.8)	492 (96.3)	269 (97.8)	
Working	13 (1.7)	13 (2.5)	0 (0)	
On official leave	2 (0.3)	1 (0.2)	1 (0.4)	
Managing household	9 (1.1)	4 (0.8)	5 (1.8)	
Unemployed	1 (0.1)	1 (0.2)	0 (0)	
Education level *, n (%)				0.321
High	248 (31.6)	162 (31.7)	86 (31.3)	
Medium	420 (53.4)	288 (56.4)	132 (48.0)	
Low	114 (14.5)	59 (11.5)	55 (20.0)	
Unanswered	4 (0.5)	2 (0.4)	2 (0.7)	
**Household income, n (%)**				0.850
<451 €	156 (19.8)	87 (17.0)	69 (25.1)	
451–903 €	316 (40.2)	198 (38.7)	118 (42.9)	
903–1354 €	186 (23.7)	130 (25.4)	56 (20.4)	
1354–1805 €	113 (14.4)	85 (16.6)	28 (10.2)	
>1805 €	0 (0)	0 (0)	0 (0)	
Unanswered	15 (1.9)	11 (2.2)	4 (1.4)	
**Marital status, n (%)**				**<0.001**
Widowed	265 (33.7)	125 (24.5)	140 (50.9)	
Married/Extramarital union	442 (56.2)	323 (63.2)	119 (43.3)	
Divorced	43 (5.5)	36 (5.3)	7 (2.5)	
Single	36 (4.6)	27 (5.3)	9 (3.3)	
**Smoking, n (%)**				**0.001**
No	699 (88.9)	440 (86.1)	259 (94.2)	
Yes, everyday	60 (7.6)	49 (9.6)	11 (4.0)	
Yes, occasionally	23 (2.9)	18 (3.5)	5 (1.8)	
Unanswered	4 (0.6)	4 (0.8)	-	
**Dietary supplements, n (%)**				0.433
No	489 (62.2)	323 (63.2)	166 (60.4)	
Yes	297 (37.8)	188 (36.8)	109 (39.6)	
**Physical activity level**				**0.003**
High	94 (12.0)	80 (15.7)	14 (5.1)	
Moderate	174 (22.1)	109 (21.3)	65 (23.6)	
Low	518 (65.9)	322 (63.0)	196 (71.3)	
**Anthropometric characteristics, mean (sd)**				
Weight	81.2 (15.3)	82.9 (15.8)	78.0 (13.8)	**<0.001**
Height	169.1 (9.5)	169.9 (9.6)	167.8 (9.2)	**0.003**
BMI	28.3 (4.4)	28.6 (4.4)	27.7 (4.2)	**0.003**
Energy reporting **, n (%)				0.470
Overreporters	1 (0.1)	1 (0.2)	0 (0.0)	
Plausible energy reporters	596 (75.8)	383 (74.9)	213 (77.5)	
Underreporters	198 (24.1)	127 (24.9)	62 (22.5)	

* International Standard Classification of Education (ISCED): Low education–ISCED levels 0–2; medium education—ISCED levels 3–4; high education—ISCED levels 5–8; ** Classification according to Goldberg cutoffs described in the EU Menu methodology [15]; *** Mann–Whitney U Test (two-group comparisons); chi-square test (sex, place of residence, supplement use), level of statistical significance at *p* < 0.05 (in bold).

**Table 2:** Daily energy and macronutrient intake in older adults in Croatia *.

	Total (N = 786)	65–74 Years (n = 511)	≥75 Years (n = 275)	*p* **
Energy (kcal/day)	1549.7 (621.9)	1628.9 (653.0)	1442.9 (557.7)	**<0.001**
Carbohydrates (g/day)	164.7 (68.2)	170.2 (69.5)	157.2 (63.4)	**0.001**
Fat (g/day)	61.8 (33.7)	64.7 (33.2)	55.3 (29.0)	**<0.001**
Protein (g/day)	58.1 (27.1)	60.0 (27.1)	53.4 (26.4)	**<0.001**
% E				
Carbohydrates	43.5 (11.9)	42.1 (12.3)	45.6 (10.6)	**<0.001**
Fat	36.4 (10.8)	37.7 (11.3)	35.2 (10.5)	**<0.001**
Protein	14.8 (4.3)	14.7 (4.2)	15.1 (4.3)	0.072

* presented as median and interquartile range (IQR); ** Mann–Whitney U Test, level of statistical significance at *p* < 0.05 (in bold).

**Table 3:** Effect of BMI and PA on daily energy and macronutrient intake in older adults *.

		PA	BMI	BMI and PA
Energy (kcal/day)	65–74 years	0.459 **	**0.048**	0.196
	≥75 years	0.050	0.336	0.101
Carbohydrates (g/day)	65–74 years	0.308	0.791	0.182
	≥75 years	**0.005**	**0.013**	**0.012**
Carbohydrates (% E)	65–74 years	0.960	**0.014**	0.941
	≥75 years	0.674	0.177	0.588
Fat (g/day)	65–74 years	0.383 **	**0.001**	0.152
	≥75 years	0.368	0.559	0.451
Fat (% E)	65–74 years	0.637	**0.003**	0.571
	≥75 years	0.660	0.104	0.599
Protein (g/day)	65–74 years	0.854	0.059	0.664
	≥75 years	0.155	0.974	0.170
Protein (% E)	65–74 years	0.807	0.986	0.783
	≥75 years	0.221	0.365	0.230

* GLM results; *p* value of main model presented, level of statistical significance at *p* < 0.05 (in bold); ** post-hoc analysis showed higher energy (*p* = 0.010) and fat intake (*p* = 0.001) in participants with a low PA level.

**Table 4:** Main food categories intake (g/day) in older adults in Croatia *.

	Total (N = 786)	65–74 Years (n = 511)	≥75 Years (n = 275)	*p* ***
Grains, grain products, and potato	209.9 (125.1)	209.7 (125.8)	210.1 (125.2)	0.482
Fruit	160.0 (188.1)	166.8 (195.3)	156.6 (174.3)	0.287
Vegetables	133.9 (102.7)	116.5 (107.4)	110.8 (99.9)	0.107
Legumes, nuts, and seeds	3.2 (28.4)	4.4 (33.4)	0.0 (23.9)	**0.033**
Meat, poultry, fish, and eggs	129.5 (108.3)	136.7 (111.9)	120.9 (98.2)	**<0.001**
Milk and dairy products	153.0 (198.4)	144.3 (213.6)	175.0 (181.3)	**0.003**
Fats and oils	18.3 (15.1)	19.0 (15.1)	16.6 (14.9)	**0.003**
Salty snacks	0.0 (0.0)	0.0 (0.0)	0.0 (0.0)	0.968
Sweets	34.5 (86.0)	38.5 (95.1)	28.0 (67.4)	**0.020**
Beverages **	1028.0 (797.2)	1087.8 (786.5)	877.4 (731.0)	**<0.001**
Alcoholic beverages	3.7 (102.1)	6.1 (109.1)	2.1 (100.0)	**<0.001**
Dietetic products	0.0 (0.0)	0.0 (0.0)	0.0 (0.0)	0.086
Miscellaneous foods	3.8 (3.0)	4.1 (3.1)	3.5 (3.0)	**0.001**

* presented as median and interquartile range (IQR); ** including water; *** Mann–Whitney U Test, level of statistical significance at *p* < 0.05 (in bold).

**Table 5:** Effect of BMI and PA on food group intake in older adults *.

		PA	BMI	BMI and PA
Grains, grain products, and potato	65–74 years	0.397	0.199	0.373
	≥75 years	0.987	0.517	0.988
Fruit	65–74 years	0.406	0.059	0.400
	≥75 years	**0.006**	**0.001**	**0.009**
Vegetables	65–74 years	0.199	0.968	0.105
	≥75 years	0.796	0.202	0.802
Legumes, nuts, and seeds	65–74 years	0.192	0.685	0.186
	≥75 years	0.226	0.943	0.224
Meat, poultry, fish, and eggs	65–74 years	0.892	**0.005**	0.963
	≥75 years	0.256	0.776	0.258
Milk and dairy products	65–74 years	0.097	0.108	0.139
	≥75 years	**0.018**	**0.006**	**0.029**
Fats and oils	65–74 years	0.956	0.424	0.928
	≥75 years	0.609	0.570	0.499
Salty snacks	65–74 years	0.909	0.743	0.884
	≥75 years	0.738	0.965	0.769
Sweets	65–74 years	0.739	0.422	0.552
	≥75 years	0.562	0.593	0.618
Beverages **	65–74 years	0.796	0.426	0.883
	≥75 years	0.529	0.713	0.598
Alcoholic beverages	65–74 years	**0.041**	0.326	0.070
	≥75 years	0.860	0.961	0.929
Dietetic products	65–74 years	0.300	0.243	0.361
	≥75 years	0.882	0.517	0.911
Miscellaneous foods	65–74 years	0.052	0.267	**0.045**
	≥75 years	0.494	0.227	0.363

* GLM results; *p* value of main model presented, level of statistical significance at *p* < 0.05 (in bold); ** including water.

## Data Availability

The data are available from the Croatian Agency for Agriculture andFood, but restrictions apply to the availability of these data. Data are available upon request and with permission of the Croatian Agency for Agriculture and Food.

## Supplementary Materials

The following supporting information can be downloaded at: https://www.mdpi.com/article/10.3390/epidemiologia7030071/s1, Table S1: The list of food categories and subcategories.

## Abbreviations

The following abbreviations are used in this manuscript:

BMI	Body mass index
PA	Physical activity
IPAQ	International Physical Activity Questionnaire
GLM	General linear model
