# Supplementary material for: Age-Related Differences in Dietary Intake and Nutritional Status Among Older Adults in Croatia: Results from a National Food Consumption Survey
Source: Epidemiologia (Basel). 2026 May 21;7(3):71. doi: 10.3390/epidemiologia7030071 (PMC13214645; doi:10.3390/epidemiologia7030071)
Supplement: Supplementary file 1 [file epidemiologia-07-00071-s001.zip › epidemiologia-4220025-supplementary.pdf]

Table S1 The list of food categories and subcategories

Food category	Food subcategory
Milk and dairy products	*Milk*<br>*Flavoured milk*<br>*Fermented dairy products*<br>*Flavoured fermented dairy products*<br>*Dairy desserts*<br>*Cheese and cheese spreads*
Fruit	*Fresh and frozen fruits*<br>*100% fruit juices*<br>*Canned fruits*<br>*Dried fruits and fruit bars (100% fruits)*
Vegetables	*Fresh, and frozen vegetables*<br>*100% vegetables juices*<br>*Canned and dried vegetables*
Grains, grain products, potatoes and tubers	*Bread, rolls and tortillas*<br>*Grains, grits and flour*<br>*Pasta*<br>*Breakfast cereals*<br>*Fresh and frozen potatoes and tubers*
Meat, poultry, fish and eggs	*Eggs*<br>*Fresh and frozen red meat*<br>*Fresh and frozen poultry*<br>*Fresh and frozen fish, seafood and products*<br>*Processed meats (red meat and poultry)*<br>*Frozen breaded meat and fish products*
Legumes, nuts and seeds	*Fresh, dried, canned and frozen legumes*<br>*Seeds and seeds products*<br>*Nuts and nuts products without added salt and sugar*<br>*Nuts and nuts products with added salt and sugar*<br>*Milk and meat alternatives*<br>*Fermented milk alternatives*
Fats	*Plant oils*<br>*Animal fats*
Sweets	*Chocolate and chocolate spreads with additives*<br>*Cocoa powder*<br>*Biscuits and dry cakes*<br>*Cakes and tarts*<br>*Marmalades, jams and jellies*<br>*Honey and sweeteners*<br>*Ice creams based on water and/or milk*<br>*Sugar*<br>*Confectionery and chewing gum*

	Other
Salty snacks	*Grain based salty snacks*<br>*Potato based salty snacks*
Beverages	*Water*<br>*Tea*<br>*Fruit and vegetable juices*<br>*Other non-alcoholic beverages*
Miscellaneous foods	*Salt*<br>*Condiment with dehydrate vegetables, spices and salt*<br>*Yeast*<br>*Dehydrated stocks*
Dietetic products	*Supplements*<br>*Enteral supplementation*<br>*Protein powders*
Beverages	*Water*<br>*Tea*<br>*Coffee*<br>*Fruit and vegetable nectars*<br>*Non-alcoholic beverages*<br>*Energy drinks*
Alcoholic beverages	*Beer*<br>*Wine*<br>*Spirits (excluding liquers)*<br>*Liquers*<br>*Liqueur wines*<br>*Other mixed acoholic beverages*<br>*Vinegar*
